# Strategic housing decisions and the evolution of urban settlements: optimality modelling and empirical application in Ulaanbaatar, Mongolia

**DOI:** 10.1098/rsos.241415

**Published:** 2024-10-30

**Authors:** Natalia Fedorova, Anne Kandler, Richard McElreath

**Affiliations:** ^1^ Department of Human Behaviour, Ecology and Culture, Max Planck Institute for Evolutionary Anthropology, Leipzig, Germany

**Keywords:** settlement strategy, residential choices, housing investments, niche construction, human behavioural ecology

## Abstract

Investments in housing influence migration and landscape construction, making them a key component of human–environment interactions. However, the strategic decision-making that builds residential landscapes is an underdeveloped area of research in evolutionary approaches to human behaviour. Our contribution to this literature is a theoretical model and an empirical test of this model using data from Ulaanbaatar, Mongolia. We develop a model of strategic housing decisions using stochastic dynamic programming (SDP) to explore the trade-offs between building, moving and saving over time, finding different trade-offs depending on optimization scenarios and housing costs. Household strategies are then estimated using data on 825 households that settled in the Ger districts of Ulaanbaatar between 1942 and 2020. The Ger districts are areas of self-built housing that feature both mobile dwellings (gers) and immobile houses (bashins). Using approximate Bayesian computation (ABC), we find the parameters of our dynamic programming model that best fit the empirical data. The model is able to capture the time horizon of housing changes and their bi-directionality, showing that moving from a fixed to mobile dwelling can also be an optimal strategy. However, the model underpredicts household persistence in dwelling types. We discuss deviations from model predictions and identify a more detailed exploration of risk and population mixes of strategies as key steps for future research.

## 1. Introduction

The human built environment has drastically changed in the past 10 000 years. To the best of our knowledge, people living before the Holocene built relatively low-investment residential structures and lived in low-density settlements. In contrast, contemporary humans are a predominantly urban species, with a majority of the human population living in cities [1]. Cities are major human investments in the built environment: large, dense, expensive to build and maintain, and ecologically dominant through their energy expenditure and dependence on a sprawling anthropogenic landscape of intensive, and today industrial, agriculture [2,3]. But cities are no more static than rural landscapes, with people in both constantly making decisions about what sort of dwellings to live in, with significant consequences for land use trajectories, mobility systems and adaptations to climate change [4–6].

Human landscapes are the products of strong feedbacks between strategic behaviour and the material products of that behaviour. Behaviour produces landscapes, and landscapes produce behaviour. Feedback with the residential landscape has been neglected both in research on the Holocene transformation, but also in studies seeking to understand contemporary human–environment interactions. In the evolutionary sciences, the built environment is most often formulated as a consequence of other choices people make about their survival. Investments humans make in residential dwellings in this view are products of the subsistence, mobility regimes and culture of their populations [7–11]. They are not causes.

It is neither novel nor controversial to say the built landscape is as much a cause of behaviour as a consequence. But explicit modelling of this feedback is lacking. This hinders both theory development and our ability to explain historical and contemporary landscapes. Interfacing with theory and its development is important, as methodological debates across the social sciences have called for a stronger engagement with theory to better deal with the pitfalls of behavioural research [12]. Understanding human behaviour using observational, and particularly cross-sectional data, can be mired in problems. Associations between variables can be spurious, or can have no causal relationship. Humans also proficiently manage their trade-offs, which can mask expected relationships in the data, leading us to make incorrect inferences. The generative inference approach formulates a workflow that can closely integrate theoretical models with empirical data, making assumptions explicit and revealing situations of equifinality [13]. We make use of this approach to conduct a workflow that can connect optimality modelling with empirical data in a robust and transparent way.

We address the lack of theory that includes explicit dynamic feedback between strategy and landscape. We build a dynamic model of household decisions over time. Decisions have consequences for durable technology that influences the landscape: households can either build fixed housing or remain in mobile dwellings. Feedback from these decisions is modelled by including housing in the model state space, such that it influences optimal behavioural trajectories. Households will thus have different behavioural trajectories based on the type of dwelling they occupy. As such, optimal strategies influence the residential landscape, and the residential landscape influences optimal strategies. Our aim is to understand how building, moving and saving behaviour can be optimally patterned over time in the context of dynamic feedbacks from the landscape.

We begin with a model that focuses on households, but the approach is quite general. Given different household goals, we solve for optimal strategies over the lifetime of each household in the presence of a stochastic environment. Different goals produce different strategies, and these in turn imply different patterns of behaviour and consequences for the landscape. After developing the model and studying its implications, we fit the model to empirical data from contemporary East Asia, estimating the model parameters that best explain the empirical patterns using a generative inference approach [13].

The models in this paper are necessarily limited. But a further contribution is to provide an explicit and fully documented example of a framework that can be expanded to address additional factors and questions. The combination of explicit dynamic and strategic models with Bayesian computation is a flexible framework for marrying generative scientific models to empirical evidence at any scale [13] (table 1).

**Table 1.** Glossary of main terms used in this study.

term	definition
behaviour	Actions available to simulated households that probabilistically lead to changes in state values. We model building, saving and moving behaviour.
final pay-off scenario	Optimization scenarios that define what households are attempting to maximize with the optimal strategy. We explore four different scenarios that take different perspectives on what elements of a household’s state space are maximized.
optimal trajectory	An optimal sequence of behaviour arising from enacting the consequences of a sequence of behaviour from the optimal strategy. It is the output of the forward simulation of the stochastic dynamic programming (SDP) model. Thus, it represents an actual optimal sequence of behaviour as taken by a simulated household.
optimal strategy	The optimal strategy represents the output of the SDP model. It consists of an optimal sequence of behaviour for each possible state configuration, held in a multi-dimensional tensor. Dimensions are state configurations (i.e. levels of states) × time steps, this yields a tensor of dimensions 2 × 2 × 2 × 3 × 40. The tensor can be indexed to find the optimal behaviour that a simulated household should enact given the state configuration they can be described by, and the time step they are in.
state	Properties of simulated households as described in the theoretical model. We model a house state (2 levels: mobile, fixed), saving state (2 levels: no savings, savings), tenure state (2 levels: no tenure, tenure), family state (3 levels: single occupant, couple, family with dependants).
state configurations	Combinations in the state space that a household can be described by, 24 in total, based on state levels (2 × 2 × 2 × 3 = 24).

## 2. Literature review

To situate the contribution of this research project, it is necessary to first explain why including the built landscape in models of human strategic behaviour is challenging, but necessary. Human settlement strategy is composed of three key decisions: where to live, for how long and what to build there. In the following paragraphs, we discuss some of the key literature that has addressed these components, emphasizing that where to live and for how long, questions relating to the mobility regimes of populations, remain disconnected from what to build there, questions of investments in the constructed landscape and how these investments influence future decisions. We thus separate the review into two sections: (i) optimal foraging and mobility regimes and (ii) technological investments and niche construction.

### 2.1. Optimal foraging and mobility regimes

Taking advantage both of the diversity of residential structures built by hunter-gatherers as well as the availability of cross-cultural data allowed researchers in the 1980s to begin connecting environmental factors, mobility regimes and the built environment. Hunter-gatherer mobility was categorized into forager and collector systems based on subsistence regimes arising from ecology [7,10,11,14]. Forager systems have high residential mobility, moving camp often, while collector systems organize around a central place, using logistic mobility to utilize the surrounding environment [11]. In relation to ecology, forager systems are predicted to arise in landscapes with more homogeneous distributions of resources, while collector systems help groups adapt to heterogeneous landscapes [8]. More recent work has formulated the forager–collector axis as a continuum on which hunter-gatherer groups can be placed, based on the number of logistic forays a group makes before returning to a central place [15].

Optimal foraging theory (OFT) has been valuable in formalizing these relationships, as OFT models make predictions of human space use by nominating what sort of resources should be pursued and in what order, thus predicting which ‘patches’ should be visited and for how long [14,16–18]. However, collecting anthropological data is an intensive exercise, and it is already difficult to collect production data that can parametrize optimal foraging models. Until recently, it was unfeasible to collect data on group and individual mobility that could address mobility predictions. But, new methods and tracking technology are enabling more focused and high-resolution research [19,20].

More recent work using agent-based modelling has also sought to formalize the relationship between environment, subsistence and mobility, showing that in populations where people move to resources, ecology is an important driver of mobility decisions. For example, Sikk and Caruso [21] use agent-based modelling to study a spatially explicit central place foraging model and its ability to predict settlement patterns, showing that resource depletion engenders mobility, and resource abundance can lead to sedentism. While we do not address changes from mobile to sedentary living in this project, we take inspiration from the optimality premise of the aforementioned literature. We place mobility alongside building and saving behaviour to better understand the strategic decisions households make about where to live and what to build there.

Cost–benefit models from population ecology based on the ideal free distribution (IFD) have been used to address how humans will settle an area, generating accurate predictions in the settling of the Channel Islands, USA [22], the spread of farming communities in Utah, USA [23] as well as the spatial organization of pastoralists in Cameroon [24]. IFD models are well suited for understanding how a population is organized in space, and how a population settles an unoccupied area, providing a distribution counterpoint to approaches focused on individual behaviour [14].

However, the above models are all based on heuristic cost–benefit foundations, with little research exploring how exactly they interact with other social and demographic processes. Work by Crema has sought to move away from simple heuristic models, constructing models that build on the IFD to consider how population dynamics interact with the costs and benefits of living together to influence settlement dynamics [25]. This approach represents a settlement-level perspective, which takes population processes seriously. However, the aforementioned approaches do not integrate feedbacks from the built environment.

### 2.2. Technological investments and niche construction

While mostly used for addressing the mobility–subsistence link, the forager–collector continuum has implications for investments in housing, postulating that reduced mobility should result in higher investments in the constructed environment. Work conducted by Diehl, Kent and Vierich [9,26,27], who sought to elucidate the relationship between a group’s mobility regime and investments in and organization of camp structures, explores the relationship between residence time and housing investments. More recently Hamilton *et al*. [28] also found that the camp structure of hunter-gatherers from 263 different groups responded to predictable dynamics between camp area, infrastructure, the number of occupants and residence time. Based on their work with the Basarwa and Bakgalagadi of Botswana, Kent and Vierich [26,27] found that the more a camp was expected to be used, the longer residents expected to stay at a specific location, the more would be invested in the residential structure. This highlights the front-loading of building decisions. A household must decide at a given time point how much to invest in a dwelling. Depending on how ‘expensive’ such a dwelling is, this decision may be trivial, or crucial. Consider the expense involved: while common residential structures built by hunter-gatherers can be built using the equivalent amount of energy of one 8 h work day [29], the per capita costs of more intensive dwellings can be extensive, particularly once we start to consider the use of industrial, global supply chains and specialist craftspeople. In other words, housing decisions can be high-stakes decisions, making it all the more necessary to situate them in time-dependent trade-off relations.

In a unique study of the mobility–ecology–settlement link, Kelly *et al.* [7] studied the settlement patterns and investments of the Mikea of Madagascar, combining within-group variation in housing type and housing investments, as connected to the social, environmental and economic situation of a people. The authors find support for certain associations between reduced mobility and housing investment, namely in more uniform and specialized building, house size and variety and more built features. However, the project also develops more nuance between ecology and housing structures, suggesting that privacy concerns in relation to sharing dynamics also inform site structure, and thus housing investments, reminding us of the need for contextualized understandings of landscape investments.

If we consider housing as a technology, it is possible to begin to formalize this relationship through the lens of the technological investment model (TIM). The TIM describes the trade-off between investment, use time and benefit or efficiency of a certain foraging technology. First versions by Ugan *et al*. [30] used the marginal value framework to identify the trade-offs between how much time was spent constructing a particular technology, how much time was spent foraging with that technology and the amount of resource that could be foraged. This formulation allows for identifying the optimal point at which a more/less costly technology will take over a less/more costly technology. Bettinger *et al*. [31] built upon this model and added different cost–benefit curves for different types of technology, to be able to address categorical shifts: what is the optimal point at which a broad category of technology will supplant a different category of technology, based on investment. We take inspiration from Mohlenhoff and Codding [32] who adapted the TIM to a patch investment framework, with the aim of elucidating when it would be optimal to engage in more elaborate niche construction. But housing, and residential investments, have particular signatures that require modifications to the TIM. Namely, optimal investments in housing need to consider trade-offs across the entire life-course, as the high investments required for some kinds of dwelling mean such buildings will only be constructed once or twice in a lifetime.

It is a point for future research to elaborate to what extent different levels of investment in the built environment influence future behaviour. Developments in niche construction theory and its applications to human systems have made strides in emphasizing feedbacks from altered ecologies [33–37]. Niche construction is an informative framework also when it comes to dwellings. Research suggests that even though building a typical hunter-gatherer residential base is not very energetically expensive, it may reduce the costs of future occupation, thus creating ‘persistent places’ in the otherwise mobile hunter-gatherer landscape [29]. Work by Haas and Kuhn [38] also suggests the material depositions of hunter-gatherers in the Lake Titicaca Basin, Peru, 7000−5000 cal BP, structured future mobility, as groups revisited locations where valuable materials were left. Aside from empirical support, research also highlights how niche construction concepts can be linked with traditional optimality models [39], suggesting the field is well prepared to integrate landscape effects into studies of human behaviour.

## 3. Analysing strategic housing investments

Our research project aims to contribute to the existing literature on human settlement strategy by conducting a study of human housing decisions and incorporating them in a framework that explicitly includes feedbacks from decisions into future behaviour. By using the appropriate modelling tools, we are able to untangle the trade-offs between mobility, housing investments and household capital. By studying different optimization goals, housing costs and their interaction, we are able to explore the optimal timings of building, moving and saving behaviour. In turn, unravelling such trade-offs means we can get at the strategic nature of housing decisions.

In order to appropriately model housing decisions, we make use of stochastic dynamic programming (SDP), a modelling technique that derives optimal strategies over time—not just optimal decisions in single time points. As detailed in the literature review above, the majority of formal models aimed at understanding the environment–behaviour link in the evolutionary human sciences are built on marginal value, i.e. cost–benefit, foundations. While this literature has been extremely productive, providing heuristic understandings of human foraging, storage and mobility (for example [8,40]), it is a technique not suited to decisions that are optimized over long time periods. When residential housing is an investment that individuals and households will make once, or at most a handful of times, in their lifetime, when they do so in relation to their life-course is crucial. It is an obvious fact to state that there are better and worse times to build a house, a point not lost on any current or aspiring home builder.

Of course, in populations where housing is lower investment, this concern is reduced, but we should still develop models that are able to handle the entire diversity of human behaviour. In this way, it will also be possible to study exactly what counts as high versus low investment when it comes to the human built environment. Moreover, research on ‘persistent places’ serves to illustrate that costs of any amount are liable to reduction where possible, so low-investment housing does not imply an absence of strategic building behaviour [29].

Optimality models have a rich and fruitful history in evolutionary studies of human behaviour [41]. Importantly, behaviour need not be optimal for optimality models to be useful. While initial formulations in forager studies made neoclassical assertions that maximizing strategies would be adaptive and thus provide a suitable assumption [42], optimality models can be viewed much more generally as generative models that make non-null predictions. That is, they make specific, directional predictions instead of focusing on a difference/lack of difference between groups. In this context, it is precisely the deviations from optimality that help us understand trade-offs in the system and thus guide theory and future investigation. A deviation from a directional prediction gives us clues about what mechanism the model may be missing. In contrast, null models provide little opportunity for scaffolding future theory development; if a null model is confirmed—the process under question is not found in the data—we have no indication of what we should explore next.

To understand household decisions, we model three potential behaviours: building, moving and saving, exploring how different optimization goals and housing costs interact with when each behaviour should occur. We derive specific sequences of behaviours over time, showing when it would be optimal to build, to save or to move to a different patch. Our choice of modelling technique also has the advantage of easily integrating stochasticity, as the derived optimal strategy responds to probabilistic pay-offs to behavioural choices. Most importantly, our model allows us to consider housing as a state, and thus implicitly include the feedbacks investments in the built environment have on future decisions. That is, because housing is coded in the state space of households, we are able to include changes dwelling types (from mobile to fixed) have on optimal behavioural trajectories.

To illustrate with an example, we can imagine a hypothetical family who chose to buy a house after having lived in apartments for a decade. Waving away some important assumptions, we can postulate that after buying the house, certain behaviours are less or more likely; the optimal trajectory of behaviour has changed. The family may be less likely to move in the near future, and more likely to invest in a large terrace. This has population-level implications: we can also imagine that the family lives in a neighbourhood that is currently experiencing an influx of families buying houses. As such, the population mix of optimal trajectories of behaviour is changing too, in conjunction with the material fabric of the neighbourhood, as it fills up with home owners. Understanding optimal trajectories in this scenario, particularly in circumstances where we do not have access to longitudinal trajectories of household behaviour, requires we explicitly model housing within the decision and state space of households. This in turn allows us to interrogate the evolution of urban environments through the residential investment choices of households, and the consequences these have for optimal behavioural trajectories at the individual and population level.

We subsequently make use of our model in analysing empirical data from Ulaanbaatar, the capital of Mongolia (figure 7*a*). Since the democratic turn in Mongolia in 1990, easing controls on internal migration as well as divesting of funds from livestock industries has resulted in a dramatic increase in Ulaanbaatar’s population, now *ca* 1.5 million. This increase has largely not been matched by increases in public housing developments. As a result, much of the outskirts of the city of Ulaanbaatar are formed by so called ‘Ger districts’ (after the Mongolian name for the vernacular mobile, felt dwellings of local pastoralists), areas of self-built sprawl with limited services and infrastructure.

Households in the Ger districts build two types of dwellings: **gers**—mobile, circular felt dwellings, or **bashins**—houses, immobile dwellings built of wood, brick or other industrially produced materials (see figure 6). This binary dwelling situation provides a rich test case in which to explore how households choose which dwelling to build, when they change between types and what consequences this has for their livelihoods and future behaviour. Because gers tend to be less expensive than bashins, the case allows us to explicitly track and explore when households decide to invest more in their dwellings. Moreover, since bashins cannot be moved, the dwelling contrast also allows us to see under which conditions households make localized and fixed investments in their environments.

We postulate that transitions to fixed and localized investments in particular patches contribute to the evolution of urban environments, by creating built-up landscapes with consistent occupation that attract other urbanization dynamics, such as infrastructure expansion and service provision. We thus focus on dwelling transitions in this study, exploring how optimal trajectories of building, saving and moving behaviour predict changes between the two different dwelling types. In this way, we address the workings of residential investment as a generative dynamic of urbanization.

The SDP model is used as a generative model to create data which are comparable with that collected in Ulaanbaatar, yielding corresponding information on housing, savings, migration histories and tenure. While the data we collected in Ulaanbaatar is cross-sectional, interfacing with the model allows us to infer most-plausible behavioural trajectories assuming optimality. In this way, we are able to formally assess the match between model and data, making use of approximate Bayesian computation (ABC) to derive parameter values of best fit.

The rest of the manuscript is organized as follows:

**Section 4: Optimal behaviour in constructed landscapes** details the SDP model. We conduct an analysis of the theoretical model, focusing on the trade-offs generated by saving, building and moving behaviour, over time. We discuss how different optimization scenarios and dwelling costs influence time-sensitive optimal strategies. The section concludes by developing a theoretical understanding of the relationship between optimal behavioural trajectories and housing transitions.

**Section 5: Empirical application** focuses on the empirical case of Ulaanbaatar. We first detail the generative pipeline: discussing how the SDP model can be used to generate data comparable to the empirical data from Ulaanbaatar. We subsequently use ABC to derive the joint posterior distribution of best-fit parameter values of the optimality model, including optimization scenarios and dwelling cost profiles. The results of this analysis allow us to reflect on the empirical situation and on the advantages and limitations of the model. This section stands independently, and includes enough information on the model for readers interested specifically in the case study.

We end the manuscript with a discussion, paying close attention to how empirical deviations from optimal strategies can inform future research.

## 4. Optimal behaviour in constructed landscapes

We construct an optimality model to explore the trade-offs in decisions about building, moving and saving behaviour. Given that housing decisions take place over time and are at the mercy of stochastic changes in households’ environments, we use SDP, following Mangel and Clark [43]. SDP allows us to go beyond heuristic, time-insensitive models of optimal behaviour and instead construct an optimal strategy through time under uncertainty [44]. While SDP is a component of the theoretical biology toolkit, it has not been extensively applied in evolutionary approaches to human behaviour. Nonetheless, exceptions demonstrate the value of this approach.

A study by Mace [45] builds a SDP model of a livestock production system. Mace uses the model to reveal optimal herd composition over time which maximizes long-term household survival. The research strategy demonstrates that maximizing short-term gains may not equal maximizing long-term survival. Similarly, Milner-Gulland *et al*. [46] build a SDP model to explore production decisions available to agropastoralists, focusing on the choice between high- and low-risk crops in relation to household wealth. SDP has also been used by Luttbeg *et al.* and Mace [47,48] to explore the demographic transition. Luttbeg *et al.* construct the dynamic state model explicitly to address marriage decisions in the Kipsigis of Kenya, with the method allowing the authors to identify a polygyny threshold in this population. On the other hand, Mace models the relationship between wealth inheritance and family size, showing that increasing child costs decreases optimal fertility and increases the wealth left to each child, providing a possible route through which fertility is reduced in populations. In each case, SDP allows for the derivation of optimal trajectories of behaviour that are sensitive to the time horizon of decisions and their consequences.

In this section, we detail how our model of housing decisions is constructed, and how insights about optimal behavioural trajectories are gleaned. We then present the results of our model analyses.

### 4.1. Model definition

The purpose of an SDP model is to derive an optimal strategy—a means to derive optimal trajectories of behaviour, under particular optimization goals and uncertainty. Our model focuses on the behaviour of households. SDP requires that we define states that describe a simulated household at any time point, behaviours that households can enact, how the defined behaviours enact changes in a household’s states and the final pay-offs accrued by households at the end of model run time.

**State space.** In our model, the state space consists of four variables: the house state (mobile or fixed house), savings state (with or without savings), tenure state (with or without tenure) and family state (a single person, a couple, a family with dependants). House, savings and tenure each have two levels, and the family state has three levels, thus yielding 24 possible state configurations of the four states, with which a simulated household can be described (see table 2). For example, one possible configuration a simulated household could be described by is living in a mobile house, with savings, no tenure and consisting of a family with dependants.

**Table 2.** SDP model parameters.

parameter	description	event successful	event unsuccessful
p_s_save	probability of gaining saving state when saving	saving state increase	no change
p_h_build	probability of gaining house state when building	house state increase	no change
p_l_move	probability of moving and gaining tenure	increase in tenure state and loss of house state	no change
build_condition	are savings required to build? (yes, no)	–	–
p_s_loss	probability of losing saving state at each time step	decrease in saving state	no change
p_force_move	probability of being forced to move, in the absence of tenure, at each time step	move and obtain tenure with probability p_l_move	no change
final pay-off	scenarios for pay-offs accrued for combinations in state space (baseline, family baseline, additive, house priority)	–	–

**Behaviours.** In each time step, a household can enact one behaviour. We model three behaviours: building, moving and saving. Each behaviour is independent from the others, i.e. building is not linked to moving, etc.

**Figure 1.**
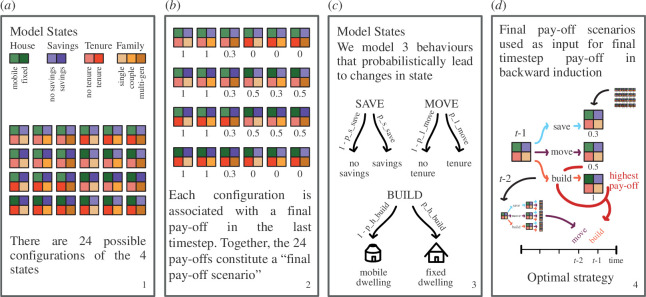
Overview of SDP model dynamics: schematic representation of how optimal strategy is calculated, as a result of interactions between final pay-off scenarios, model behaviours and the state space. Panel (*a*) visualizes the SDP model state space. Panel (*b*) illustrates how final pay-off scenarios relate to state space configurations. Panel (*c*) describes the three behaviours we model. Panel (*d*) visualizes the backward induction algorithm that calculates the tensor of optimal strategies.

**State changes.** Each behaviour is associated with a probability with which it can enact changes in a household’s state(s). Saving is associated with probability p_s_save, which defines how likely saving behaviour is to lead to an increase in the saving state (i.e. how likely saving is to be ‘successful’). If saving behaviour is ‘successful’, it results in an increase in the saving state (the saving state changes from without to with savings). Moving is associated with p_l_move; with probability p_l_move, moving behaviour is ‘successful’ and results in a household moving and obtaining tenure (the tenure state changes from no tenure to with tenure). If moving is ‘unsuccessful’, the household does not move and does not obtain tenure (no change to the tenure state). Note, however, that our model is not spatial, and thus moving is purely conceptual. Building is associated with p_h_build; if building is ‘successful’ the household builds a fixed house (the house state changes from mobile to fixed), if building is unsuccessful the house state does not change. By including housing in the state space, we derive the optimal strategy accounting for changes in the residential environment. There is no behaviour that directly results in the increase of family state; instead this is achieved through the other states. Family state increase is more likely for agents with houses and tenure and no savings, as we postulate that saving in current time takes resources away from family formation, while housing and tenure tend to provide long-term capital. Since the saving state is only binary, the effect is not cumulative, but rather saving in t−1 is associated with a reduction in the probability of family state increase in time step t. Increases in the family state are thus brough about indirectly by the consequences of building, moving and saving. See table 2 for a list and explanation of all model parameters and figure 1 for a schematic representation of the SDP model.

**Figure 2.**
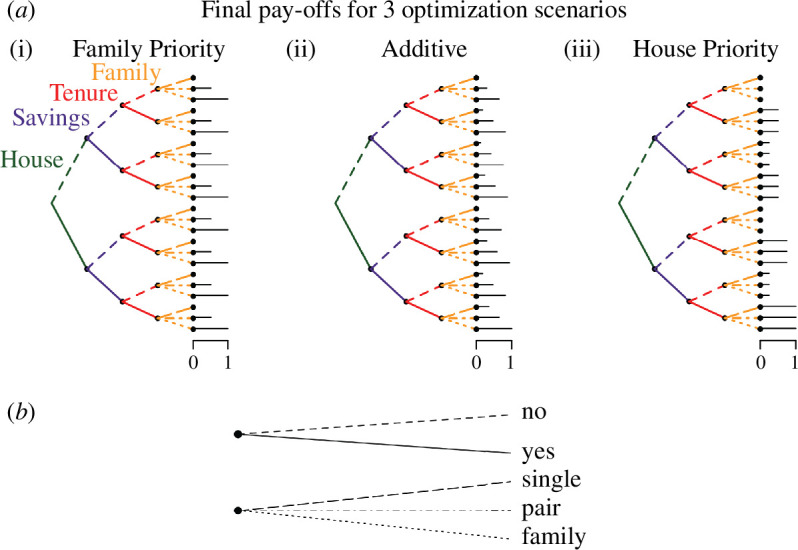
Final pay-off visualizations. (*a*) A visualization of three final pay-off scenarios. Each tree reflects how different combinations in the parameter space are rewarded at the end of time (black bars), here scaled to be between 0 and 1. Sections of the tree represent parameters and their values (house state (yes or no), saving state (yes or no), tenure state (yes or no) and family state (single, pair, family with dependants)). (*b*) The relationship of line segments with state space: for binary states, dashed lines going upwards denote not having that state (i.e. mobile dwelling for house state, no tenure for tenure state and no savings for saving state). For family state, lines denote the three possible values: single, couple, family with dependants.

**Final pay-offs.** Final pay-offs influence pay-offs accrued at the end of model run time, depending on which state configuration a household occupies (see figure 2). We construct the model to represent an adult lifespan, to mirror the period in which households may actively be making housing and mobility decisions. Defining the final pay-offs requires considering what human beings may be optimizing over their lifetime. Within the evolutionary sciences, this is an open question, as research connecting decision-making with lineage growth, or fitness, is still lacking [49,50]. As such, we do not make evolutionary claims, and do not postulate pay-offs with direct impacts to fitness. Rather, we aim to understand how sensitive behavioural trajectories are to optimizing for different aspects of the state space.

To this end, we *a priori* develop four final pay-off scenarios that offer different optimization perspectives, starting simple and then postulating possible combinations. We test a baseline scenario (material baseline) to check the model is working as expected, before testing three further scenarios where we disentangle different relationships between savings, tenure, housing and family.

The first final pay-off scenario we test is a material baseline, where only savings and tenure are optimized. This means that increasing saving state and increasing tenure state ends with the highest pay-off at the end of running time. The results of this scenario are presented only in the electronic supplementary material, as they fulfil our logic check—in this scenario, no households build (§5). The second scenario is ‘family priority’, here the state being optimized is the family state, so agents achieve highest pay-offs if they maximize their family state. Next, we test a pay-off scenario dubbed ‘additive’ where each state matters equally, and the highest pay-off is achieved when all states are maxed out. That is, increases in each state add the same benefit. The final scenario we test is dubbed ‘house priority’; maxing out the house state leads to the highest pay-off, but there is some sensitivity to risk, in the sense that the highest pay-off also requires tenure and savings, while having a house but no tenure or savings leads to a lower pay-off. In effect, the house priority scenario differs from the additive scenario mainly in removing the effect of the family state. We visualize the final pay-offs in figure 2*a* in the form of trees. These trees are not decision trees, rather reflecting how each of the 24 combinations in the four-dimensional state space is rewarded at the end of model running time.

SDP allows us to include stochasticity in the dynamic, as the consequences of behaviours are probabilistic. Additionally, we include loss of savings, and moving at a suboptimal time as stochastic shocks. Each is accompanied by a probability (p_s_loss and p_force_move, respectively (see table 2)) that determines whether a household loses savings, or is forced to move, in each time step t.

We also code a binary parameter, building condition (BC), which defines whether agents need savings to build a house or not. In effect, BC defines whether building a fixed dwelling also means building a higher-investment dwelling, or whether the two dwelling forms have the same cost.

When all is coded and defined, the SDP model algorithm works backward to calculate the optimal strategy for each configuration in the state space, to achieve the highest possible final pay-off. That is, the backward induction starts at a state space configuration, and calculates what behaviour at t−1 would have led to the highest pay-off. This calculation depends on the probabilities associated with each behaviour for enacting changes in the state space, because those define the success likelihoods of each behaviour and thus the potential of each behaviour to lead to a change in a relevant state. Importantly, if all three behaviours have the same pay-off, saving is prioritized. The outcome of the SDP calculation is thus a multi-dimensional tensor holding a sequence of behaviour (save, move, build) of the length of model run time, for each of the 24 combinations in the state space.

**Figure 3.**
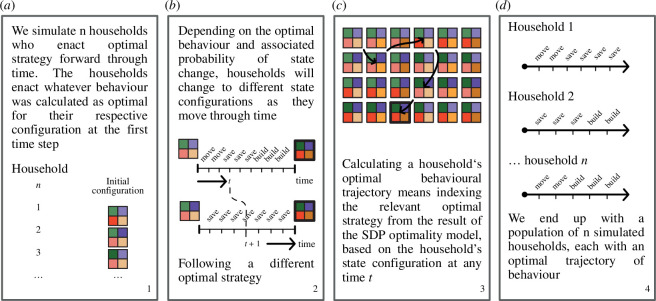
Overview of forward simulation dynamics: schematic overview of how households are simulated into the state space and enact optimal strategies and their consequences to formulate optimal behavioural trajectories. Panel (*a*) describes the initialization of the forward simulation. Panels (*b* and *c*) describe how a simulated household will access a relevant optimal strategy. This is important for how interactions between behaviour and the state space are characterized in our model, as the state space configuration (and for landscape feedback the house state specifically) occupied by a household will influence the optimal strategy that is enacted, just as the optimal strategy influences the occupied state space configuration. Panel (*d*) visualizes the longitudinal data that are generated by the forward simulation.

An optimal strategy defines the best action for any possible state configuration of a household at every possible time point, but not the consequences of those decisions, and thus not the changes in state configurations that an agent enacting the optimal strategy would experience. Moreover, a multi-dimensional tensor of optimal behavioural sequences does not lend itself to straightforward analysis. We thus construct a stochastic simulation which runs forward through time (see figure 3 for a schematic overview). This simulation is structurally equivalent to the optimality model, but now agents are simulated to enact the behaviours of the optimal strategy and incorporate their consequences. Agents are randomly simulated into the state space, and as model time runs forward, they access relevant optimal strategies, based on the time step t and the state space configuration they occupy. Based on the modelled behaviours and their associated probabilities, in t+1, an agent household may now be described by a different state configuration, now having to access a different part of the optimal strategy to enact. For more information on how optimal behaviour is calculated and accessed in the forward simulation, see electronic supplementary material, S1. The output of the forward simulation is an optimal trajectory of behaviour for each simulated household.

### 4.2. Model analysis

To understand the mechanics of the model, and reveal how optimal trajectories of behaviour respond to different parameter values, we run a parameter sweep, following standard practice [51]. That is, we observe how the optimal trajectories of behaviour change in response to isolated changes to each parameter to understand the influence of each parameter on the overall behavioural trajectory. To do so, we generate 1944 combinations from the parameter space, inputting three different values, 0.25, 0.5 and 0.75 for each of the five probability parameters, with two different values for the build condition and the four different pay-off scenarios. We generate synthetic data for 100 agents, over 40 time steps, running each parameter combination 10 times. We run the model to 40 time steps to reflect an adult lifespan. That is, we postulate that people start making their own housing decisions in adulthood, and continue to do so until old age. Simulation checks also give us confidence that this time interval accurately captures the behavioural dynamics present in the model.

The results of the parameter sweep are detailed in electronic supplementary material, S2.

**Figure 4.**
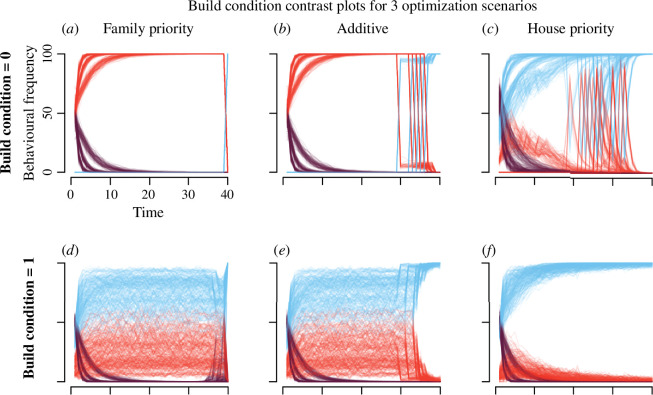
When is it optimal to build, move and save? Optimal strategy results of the parameter sweep: behavioural frequency outcomes for different optimization scenarios (family priority, panels (*a* and *d*); additive, panels (*b* and *e*); house priority, panels (*c* and *f*)) and build condition (whether savings are required for building, panels (*d*, *e* and *f*), or not, panels (*a*, *b* and *c*)). Each parameter combination yields three lines—the behavioural frequencies of building, saving and moving over time. At each time point *t* on the x axis, we can read for how many agents it was optimal to try to build, move or save, out of a 100. Note this does not imply the behaviour was ‘successful’ (e.g. moving resulted in tenure). The number of lines in total is the number of lines from the parameter sweep fulfilling the specific optimization scenario and build condition.

We visualize a subset of the parameter sweep in figure 4. Here, we focus on showing variation in optimal behavioural trajectories based on three different pay-off scenarios (family priority, additive and house priority), and the two values of build condition (are savings needed to build a house or not). In figure 4, lines represent frequencies of the three possible behaviours (blue for saving, red for building and purple for moving)—how many households out of the 100 simulated do which behaviour at each time step. Each parameter combination run yields one line for each behaviour.

The consequences of the optimal strategy could be explored in a variety of ways. Since we are interested in when and why households might build higher-investment dwellings, that constitute localized investments in particular locations, we focus on changes in the house state. The house state represents changes from mobile to fixed housing, representing housing that can be taken with the household to a new location, and housing that would need to be abandoned at relocation, respectively, enacting different pressures on the optimal behaviour of households. We also consider the transition from mobile to fixed housing under different cost profiles through the build condition, are savings required to build a fixed house or not? This allows us to explore the role of housing costs in household housing decisions.

**Figure 5.**
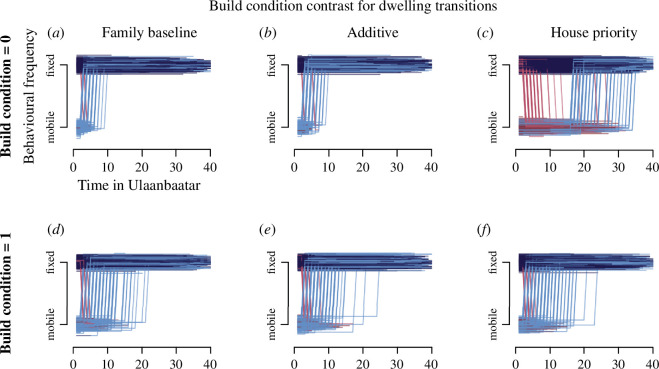
Implications of optimal behavioural trajectories for changes in the house state: Plots show dwelling transitions under different final pay-off and cost scenarios. Panels (*a* and *d*) show results for the family baseline scenario, panels (*b* and *e*) for the additive scenario and panels (*c* and *f*) for the house priority scenario. Panels (*a*, *b* and *c*) show house state changes when savings are not required to build, while panels (*d*, *e* and *f*) show results for when savings are required. Each plot shows four categories of transition: mobile-mobile (dark red), mobile-fixed (light blue), fixed-mobile (light red), fixed-fixed (dark blue). A dwelling transition implies it was optimal for an agent to build, and that building was successful. Each line represents an agent, moving through time, and agents are jittered so that thicker blocks represent many households. The x-axis of each plot represents time spent in the environment, while the y-axis represents the type of dwelling a household is in, mobile or fixed.

We construct transitions plots (figure 5 and figure 10*a*) to visualize how agents shift dwelling types over time. In the transition plots, each line is an agent, coloured by the type of transition. Moving up means building a house, while moving down means moving to a mobile dwelling.

All code is available in this github repository: https://github.com/Naty-fedorova/strategic_housing_investments.

More than a static result, the SDP model is a tool for querying trade-offs in particular areas of the state space. As such, part of the output of this manuscript is a flexible script that allows readers to generate optimal strategies under any parameter values. This script is available at https://github.com/Naty-fedorova/strategic_housing_investments/blob/main/Run_analysis/sdp_explore.Rmd.

### 4.3. Model results

In this section, we aim to discuss parts of the parameter and state space that reveal key differences and trade-offs in the optimal behavioural trajectories of households.

**Family priority scenario.** The first pay-off scenario we test is ‘family priority’, where modelled households only care about increasing their family state. The pay-off structure is illustrated in figure 2*a*(i). As detailed in the methods, there is no direct behaviour for increasing family state in the model, instead, having a fixed house and tenure positively impacts the probability of increasing family state, while having savings decreases it. In figure 4*a*, we can see that no modelled households save when savings are not required for building a fixed house. This is because savings decrease the chances of family state increase. Instead households build and move early on (in the first 10 time steps). In figure 4*d*, the build condition is 1, which means savings are required for building. Here, saving behaviour dominates—most agents save throughout model run time. How much saving occurs is affected by p_s_save and p_s_loss, the two parameters encoding how likely it is to acquire savings when saving. Interestingly, as visualized in figure 4*d*, there is an increase in moving towards the end of the time span when BC = 1, that is matched by a decrease in savings. This reflects a last attempt to acquire tenure in an effort to increase the odds of family state increase. The switch in saving and building behaviour in BC = 0 is due to all three behaviours having the same pay-off at time step 39, and so saving is prioritized for all agents. The optimal trajectory in the family priority scenario favours moving and building early on, and saving only if saving is necessary for building.

**Additive scenario.** The next pay-off scenario we explore is dubbed ‘additive’, here the best pay-off is achieved if modelled households maximize all states, as reflected in figure 2*a*(ii). In this scenario, we see a similar pattern emerge in the first 10 time steps as with the family priority scenario: households save only if savings are required for building. This can be seen by contrasting between panels (*b* and *e)* in figure 4. However, in the last 10 time steps, most households save even in the BC = 0 condition (*b*). Once households have had a chance to increase family state, through building and acquiring tenure, they will then save, as saving is also rewarded by the final pay-offs in the additive scenario. When exactly the fall-off of building behaviour happens is down to the ability to accrue savings, the harder it is, the sooner building falls off. In figure 4*b*, this leads to building and saving behaviour crossing over, as the number of households building decreases and the number of households saving increases. Saving also increases in the last quarter of figure 4*e*. In the additive scenario, a trade-off emerges between saving and building through the family state. Households save because saving is rewarded by the final pay-offs of the additive scenario, but so are increases to family state, which are adversely affected by savings. The trade-off is managed through the order of behaviours: when savings are not required for building, no saving occurs early on and households build and move to increase also their chances of family state increases. Households thus postpone saving behaviour, making it the last state to be maximized. This is true also in figure 4*e*, but since savings are required for building in these results, saving behaviour dominates throughout model run time.

**House priority scenario.** Finally, we test a scenario dubbed ‘house priority’, where maximizing house state has the best pay-offs, but tenure and savings also matter, with the second-best pay-offs arising from a combination of house and tenure state maximization, visualized in figure 2*a*(iii). This scenario allows us to look at an additive situation in the absence of the effect of family, as the highest pay-offs are family insensitive. Paradoxically, in this scenario we see less building and more saving behaviour, as shown in figure 4*c*,*f*. It is precisely because the final pay-offs are family state insensitive that saving behaviour takes off, since the cost to the increase in family state is no longer a pressure. When savings are required for building, the optimal strategy thus suggests most households save, with moving and building behaviour occurring less, and primarily in the early time steps (figure 4*f*).

However, a trade-off between saving and building still exists in the house priority scenario when savings are not required for building a fixed house, as visualized in figure 4*c*. In the house priority scenario, the trade-off operates through the tenure state, as the higher pay-offs for building are linked to having tenure. Whether building or saving takes precedence, and when, is determined by parameters p_h_build, p_s_save and p_s_loss which affect the probabilities of the outcomes of building and saving behaviour, respectively. Stochasticity plays a dominant role in the saving–building trade-off. This can be seen in the spikes of building behaviour in figure 4*c*. While not completely clear from figure 4, the spikes are total, in the sense that the frequency of building beforehand is zero in those model runs; no households build. This effect is dependent on the probability of acquiring a house when building (the parameter p_h_build). If p_h_build is low, building behaviour takes off early, peaking as mobility starts to decrease. However, as certainty increases, when p_h_build is higher or equal to 0.5, we see spiking in building frequency in later time steps (see electronic supplementary material, S2 for the full parameter sweep). As the certainty of gaining a house by building increases, households wait longer to build, waiting to acquire tenure and savings, as this is the maximal final pay-off in this scenario. However, when the certainty of gaining a house is very low, then it is optimal to rather maximize savings, and thus decrease the frequency of building. The bifurcation between savings and building caused by tenure results in the build condition being relatively less important in this final pay-off scenario.

### 4.4. Optimality implications for house state transitions

Our research focus is to unpack some of what may be driving transitions between different types of dwellings. To this end, we focus on transitions in the house state—do agents stay in the dwelling type that they start with, be it mobile or fixed, or do they change?

Generated outputs from the SDP model can be reformulated in terms of dwelling transitions, by looking at changes in the house state specifically. Seen in this way, we can interpret the optimal strategy explicitly in light of what sort of dwelling (mobile or fixed) a simulated household occupies.

Figure 5 echoes figure 4 in structure, keeping to the same contrast between BC = 0 and 1, and showing results for three final pay-off scenarios. For the family priority pay-off condition, figure 5*a*,*d*, the contrast reveals that requiring savings for building increases the number of transitions from a mobile to a fixed dwelling. More saliently, it also extends the amount of time agents spend in mobile dwellings before transitioning from mobile to fixed houses.

Despite having different optimal behaviour implications (figure 4*b*,*e* as opposed to figure 4*a*,*d*), the dwelling transition results in the additive pay-off condition (figure 5*b*,*e*), are essentially indistinguishable to family priority (figure 5*a*,*d*). However, in the family priority scenario, households remain in mobile dwellings in the BC = 1 condition longer. This reflects the premium on savings, as agents have to trade off between keeping savings or spending them on building a fixed house, forcing a trade-off between saving and building.

Figure 5*c* highlights a paradox, why is it that in a pay-off condition that rewards house building and under BC = 0, where savings are not required to build, do we see the most transitioning from a fixed to a mobile house, i.e. a loss of house state? The house priority pay-off condition serves to disentangle the effect of house and family on the optimal strategy. Through the house state, we can see that agents, optimizing for savings and tenure, move and save early on, even at the expense of losing a fixed house. The final pay-off value of tenure is responsible for this, because the value of a fixed house is tied to tenure in this scenario, it means the final pay-offs will favour losing house state in order to gain tenure, which can only be achieved through moving in our model. The trade-off between building and saving as modulated by tenure largely disappears when savings are required for building, as graph *f* in figure 5 does not differ from graph *e* or *d*. If you require savings to build, households must save, and build as soon as they having the means to do so. The broad result from figure 5 is that whether savings are required or not for building a fixed house, and so whether a fixed house is costly or not, is the main determinant of when households change dwelling types.

It is important to note that figure 5 is produced for generated populations of agents that have varying lengths of stay in the environment. As such, we do not observe households at the end of their time span, but rather a mix of stay lengths. This is why we still observe agents in mobile housing; simulating a population of agents who all stay in the environment for 40 time steps shows that every agent eventually transitions to a fixed house (electronic supplementary material, S3). In figure 5*c*, this is counter-weighted by a population of agents that continue to stay in mobile dwellings for many time spans, as it is optimal for them to maximize tenure and savings rather than tenure and housing.

### 4.5. Model discussion

Analysing the SDP optimality model reveals the importance of time in how building, moving and saving trade off. Moving and building trade-off because moving entails the loss of a fixed house. The trade-off is thus modulated through the cost of housing, as losing house state in scenarios where savings are not required to build is less problematic than when savings are required. This result reflects the time-insensitive formulation of the TIM, as the ability to save, to accrue resources for building, has important consequences for when building occurs [31]. As such, understanding the costs of different types of dwellings is a necessary first step in understanding the building decisions households make.

Saving and building also trade-off through two distinct pathways. Firstly, savings are detrimental to increases in family state but required for increases in house state, which in turn positively effects family state increases. A trade-off is thus forged between saving and building, through the family state. Whether the family state is being optimized in the final pay-offs thus has deterministic effects on whether saving or building takes precedence, and importantly, in what order. When family state increases matter, saving is favoured only when it is necessary for house building, otherwise it is minimized.

Secondly, the house priority pay-off scenario allows us to explore a saving/building trade-off as modulated by tenure. In this scenario, the trade-off is defined not by the dynamics of the family state, but rather through the tenure state in the final pay-offs, where the best possible pay-off is a combination of house, savings and tenure, while the second best is of house and tenure. The precondition of tenure allows for stochasticity to play a much more important role than in other final pay-off scenarios. The optimal strategy is more dependent on how likely it is that house state will increase when building. As such, the certainty of getting a house when building allows for the postponement of building behaviour, in favour of maximizing savings and tenure states.

Models in evolutionary approaches to behaviour tend to be limited by their shallow treatment of risk and uncertainty, which research has identified as having important impacts on optimal strategies [52–54]. The SDP modelling approach allows us to derive risk-sensitive optimal strategies, which the house priority scenario shows can have important consequences for optimal trajectories. That being said, it is important to acknowledge that the model studied here does not pull apart different ways that risk can influence behaviour.

Specifically, our exploration of risk is difficult to explicitly relate to bet-hedging dynamics. Bet-hedging reflects a dual strategy in which organisms maximize gains in favourable environments while minimizing variance in unfavourable environments. Bet-hedging is well formulated in theoretical biology [55] and has also been applied to human systems. Allen [56] has used it as a theoretical framework to consider variance minimizing and output maximizing strategies in relation to agricultural practices during Hawaiian history. Also, it has been used by Jones [57] to explain the role of risk in primate life-history. The way risk has been conceptualized in this study is within generations, as we have not modelled an evolutionary dynamic. Moreover, additional analyses would be required to pull apart the relationship between mean pay-offs (i.e. fitness in this model) and variance in pay-offs, across individuals. This would be necessary to understand how exactly optimal strategies reduce pay-off variance, and whether they do so at the level of the individual or in the correlation between individuals or a combination of both.

In this research project, we have focused on dwelling transitions, looking essentially at two cases based on cost. When savings are required, the transition between the dwelling types is one from low to high investment, whereas when savings are not required, it is a transition from mobile to fixed dwellings, without the object of cost. This dynamic allows us to see that saving takes precedence when it is required for building, with consequences for optimal balances between moving, saving and building over time, in relation to residential ecology.

Considering dwelling transitions, the model results reveal that while different model scenarios result in different optimal trajectories of behaviour, these differences are minimized and even disappear when viewed solely through changes in the house state. That is, different optimization and cost scenarios lead to less differences in house state change trajectories; they produce trajectories that cannot reliably be distinguished. As such, inferring optimal scenarios solely from the house state will lead to circumstances of equifinality; it will not be possible to distinguish between alternative optimization and cost scenarios based only on dwelling transitions. The model results thus also suggest more generally that careful and validated methods are needed when attempting to infer behaviour solely from changes in the residential landscape.

Secondly, viewing optimal strategies through the house state change serves to again emphasize the bi-directional nature of dwelling transitions. This indicates that losing house state can be optimal. Specifically, losing house state can be optimal early on in time, when high probabilities in gaining house state and conflicting optimization needs may make it the best choice to move and abandon a fixed house than to stay on a plot with no tenure. The defining role of tenure in relation to optimally losing house state also highlights that in the absence of information on tenure and land rights, drawing conclusions from housing investments for intended stay can be misguided.

The model we have constructed is clearly a major simplification of any housing situation families face in the real world. However, it proves illustrative in emphasizing the need to account for the time dimension in housing decisions. It also identifies empirical blind spots. Addressing dwelling transitions should build on the costs and outcomes of each dwelling type. Finally, the model presented here embeds feedbacks from the built environment, by making dwelling choices part of the state space. However, much more can be done to unpack the actual consequences of the house state on other parts of the state space. Here, we have focused on dwelling transitions, but the same modelling framework could be used to address how staying, or leaving, each dwelling type affects the long-term optimal strategy of households.

The way household behaviour is played out in this project ignores the presence of other households, and treats space as an infinite resource. Previous modelling work on the IFD in particular has identified important game-theoretic components of the human use of space [14], highlighting limits in our approach. Moreover, the environment is of course finite and research has shown that this finiteness itself can influence mobility regimes, and thus housing choices. Rosenberg’s [58] model of sedenterization suggests that as an environment fills up, demographically speaking, mobility becomes curtailed, creating a sedenterization feedback loop. As such, our modelling approach represents a case where such constraints must be minimized.

However, we would like to conclude the theoretical model section by stressing that the modelling framework described here can be expanded to account for settlement-level and inter-specific properties of the environment, such as density, or the behavioural trajectories of relevant others, all through expanding the state space. Such future work could expand our understanding of urbanization processes by giving us robust expectations, under the assumption of optimality, to guide our research.

## 5. Empirical application: the Ger districts of Ulaanbaatar, Mongolia

In this section, we introduce the settlement situation in the Ger districts of Ulaanbaatar and develop an analysis of dwelling transitions based on the theoretical model presented in §4. For an overview of the model dynamics, and the states and behaviours we model, readers can refer to figures 1 and 3.

### 5.1. Study area, population and empirical data

**Figure 6.**
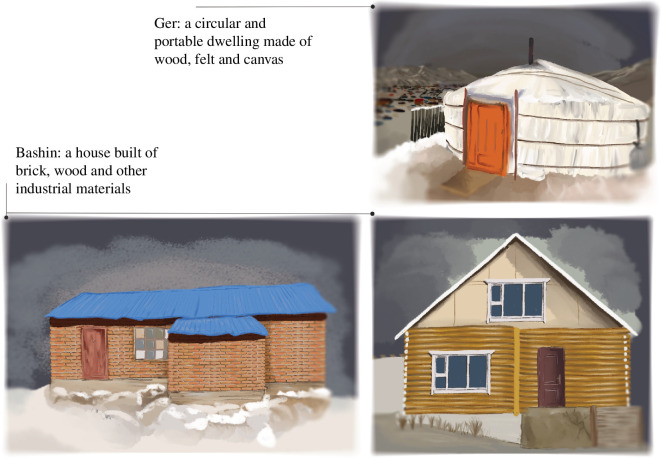
Types of dwellings present in Ger districts of Ulaanbaatar.

**Figure 7.**
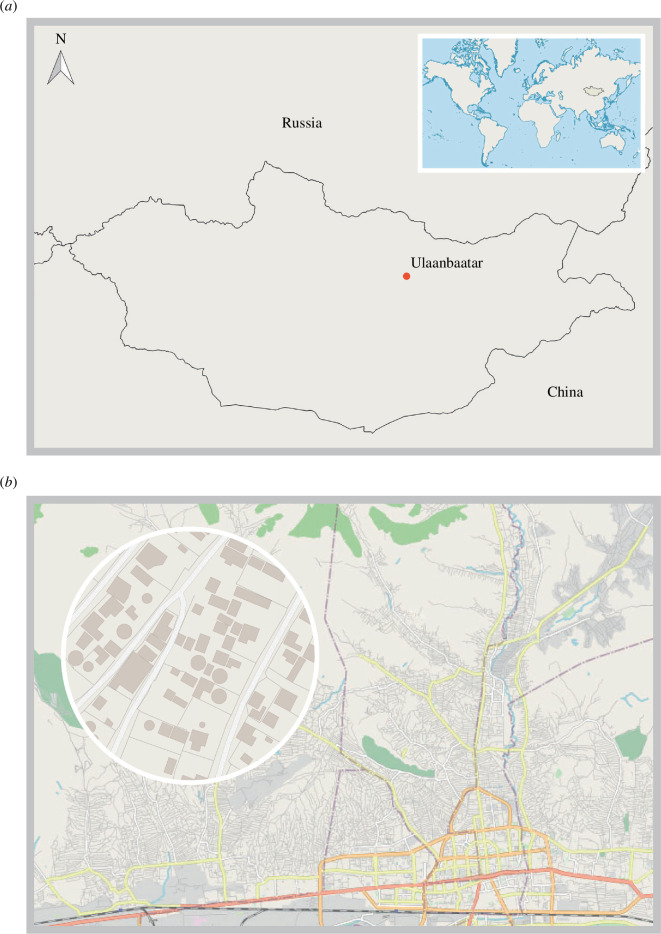
Locations of Mongolia and Ger districts. (*a*) Map of Mongolia. (*b*) Map of central and northwest Ulaanbaatar. The close-up region illustrates the built-up structure of the ger districts. Circular shapes are gers, quadrangle shapes are bashins while outlines represent land plots.

Ger districts are areas surrounding Ulaanbaatar’s formally planned and constructed core that are mainly built by households that have moved to Ulaanbaatar since the end of the socialist regime in 1990 [59]. See figures 6 and 7*b* for a map of the Northern Ger districts. Some Ger districts have much longer histories, and it is prudent to say that the general structure is present in urban environments across Mongolia and shares similarities with historical settlement arrangements from the end of the eighteenth century. It is thus incorrect to frame the Ger districts as an urban form arising out of the late twentieth century, rather the time depth and geographical spread highlight this as a form of Mongolian urban vernacular [60].

The Ger districts of Ulaanbaatar are characterized by self-built housing and a lack of amenities, services and infrastructure. The provision of centralized services increases closer to the core of Ulaanbaatar, and is also associated with the length of time that particular areas have been settled. Despite these characteristics, Ger districts do not meet the criteria of informal settlement [61]. As Byambadorj *et al*. [61] detail, despite the lack of services, Ger districts are attractive residential areas as they can be well located in relation to jobs and offer more space than apartments. Moreover, apartments in Ulaanbaatar are of low quality and offer little hope for remodelling. Finally, the Ger districts are set apart from informal settlements across the world most explicitly due to the legal infrastructure in place that allows inhabitants to officially own the land that they settle.

By some estimates, around 60% of Ulaanbaatar’s population lives in Ger districts [62]. During the socialist period, internal migration was highly regulated, and even so central housing provision was not able to meet demand in Ulaanbaatar [63]. Ger district expansion was thus slow before the revolution but deregulation of migration and the possibility to own land opened the doors of Ulaanbaatar in the 1990s [61].

Opening doors does nothing unless people actually want to move somewhere, so what are the reasons behind Ulaanbaatar’s explosive growth? A steady decline of support for rural livelihoods as well as increasing climatic instability are most often cited reasons [64,65]. A majority of Mongolia’s rural population engages in nomadic pastoralism. During the communist period, this system was restructured away from the management of kin groups to the state, and after neoliberalization, it more closely resembles ranching, as the pressure to produce meat for sale trumps subsistence pastoralism. The pastoral system has also become more unequal, with a small group of livestock owners managing the majority of livestock [66]. Part of neoliberal development has also been a dramatic decrease in the provision of rural services, such as veterinary care and winter fodder, with consequences for the rural employment landscape, but also the scaling down of rural schools and broader education and medical services. Endicott [66] explores Mongolian history from the thirteenth century to the present through the lens of land use, documenting the process of risk-management change that underpins the shifting relations between land and people at play in Mongolia today.

Dzud, summer droughts followed by severe winter events, are often cited as the main causes for herders to eventually move to Ulaabaatar. Dzud events can kill entire herds, decimating livelihoods, and have occurred more frequently in the past few decades. However, it is important to situate this in the context of little state-provided risk management. As such, urbanization in Mongolia is not simply the result of climatic change, but rather represents a pattern all too familiar elsewhere in the world; a process of state finance reorganization and redistribution such that cities are favoured and rural enterprise unsupported. Reflecting this point, research by the International Organization for Migration (IOM) finds that most households moving to Ulaanbaatar cite employment and education opportunities as main attractors [65].

While many migrants coming to Ulaanbaatar do come from herder backgrounds, the population is more diverse than common narratives allow [65]. Many inhabitants come from Soum centres or other cities in Mongolia, with already some experience with urban environments. Likewise, parts of the Ger districts represent suburban living. While most young people in the Ger districts desire apartment life, much of the housing in Ulaanbaatar is of low quality, and many choose to return to the Ger districts when older, and particularly with children or other dependants that increase space needs.

Pollution is a key issue in Ulaanbaatar. As the coldest capital in the world, where winter night temperatures regularly plummet below −30°C, heating is critical. Until recently, the domestic dwellings in the Ger districts were heated exclusively with coal burned in domestic stoves [67]. Since 2019, there has been a coal ban and the situation has improved. The coal ban was accompanied by a migration ban, in an effort to limit the swelling of the Ger districts which were pinpointed as the sources of pollution. Unsurprisingly, this ban did little to limit numbers of incoming migrants, and made it difficult for them to access already scarce local services (i.e. no public school or healthcare until registration) [65,68]. We mention this policy situation as it overlaps with the data collection period of the study.

Finally, a few words on how the actual settling process of the Ger districts occurred and continues to occur. After regime change, Mongolia had to grapple with how to privatize land. Land reform from 2003 allows each Mongolian to privatize 0.07 hectares of land in Ulaanbaatar, or bigger plots outside the capital [61]. Byambadorj *et al*. [61] provide a thorough and well-contextualized review of land reform in Mongolia since the 1990s, emphasizing the uniqueness of the Mongolian case in the post-socialist space, given the nomadic nature of the population’s history.

Families incoming into Ulaanbaatar must find an available plot of land (a khashaa), building a ger or house and a fence, and then register this plot as theirs. A secondary market for land is starting to emerge even in newer Ger districts, as land has become scarce. Newcomers will often choose to build gers on unsuitable, dangerous plots at risk of landslides and without road provision until they are able to find a suitable plot. Families, even after building bashins, will leave gers on their plots for visitors to stay in, and it is not uncommon to see plots with several gers. Despite the possibilities of land privatization, securing land can be challenging, particularly now with little land available, and the legal requirements can be obscured from newcomers, making it tricky to settle and start a life in Ulaanbaatar [69].

**Data collection.** The study detailed in this manuscript was conducted in Songhinokhairkhan khoroo (district) of Ulaanbaatar, an area of almost exclusive Ger district located North West of the city centre of Ulaanbaatar. Kheseg, or sub-districts, of Songhinokhairkhan were selected so as to maximize the variation in age of ger district settlement, following a main road from just outside the city core to the outskirts.

Data collection was conducted through a survey produced by N.F. in collaboration with kheseg leaders and Munkh-Orgil Lkhava, who was employed as the local project coordinator. Kheseg leaders hold voluntary positions with local government whereby they are responsible for the population of a kheseg, conducting work such as helping households fill in paperwork, providing advice related to government, and helping newcomers settle in. They are thus invaluable collaborators when working in the ger districts. The survey was supplemented with more in-depth interviews and participant observation conducted by N.F. over three months in 2018–2020.

A household survey was used to collect information on household demographics, migration history and investments in housing. Households were delineated by residential dwellings, and demographic information collected for all individuals. Recall was used to collect household level migration histories. Surveys were conducted by 35 kheseg leaders. More information on how the survey was developed, deployed and updated is provided in electronic supplementary material, S4.

In total, household survey interviews were completed by 1158 families.

**Empirical data.** Household surveys were transcribed and processed with the help of the data provenance group at MPI EVA (Max Planck Institute for Evolutionary Anthropology).

For the analysis in this manuscript, a subset of this database was used in which information was present for each of the variables of interest. This yielded a sample size of 825 households. We do not postulate non-random patterns of missingness that would be related to our variables of interest.

**Variables.** In this analysis, we consider a set of variables that mirror the state space considered in the SDP model.

**Time in Ulaanbaatar.** How long has a household lived in Ulaanbaatar? We used recall interviews to collect information on the residential history of each household. Respondents were asked to list locations the family had resided in. We construct a variable of time in Ulaanbaatar based on the first residential move to the Ger districts. The range of time in Ulaanbaatar is from 1942 to 2020.

**Savings.** Questions relating to money took time to fine-tune and so were presented differently in the two waves of the survey. In the second wave, direct questions about savings were asked, and so a yes/no variable was readily available. However, for the first wave, a yes/no savings variable was constructed based on the income structure of the family, if the family was not able to save from income, and had no other sources of income than the salary of members, they were listed as having no savings.

**Table:** 

no savings	have savings
521	304

**Tenure and residential moves.** The collected recalled residential history could be used to reveal household mobility, as well as dwellings at each of these locations.

**Table:** 

no tenure	have tenure
247	578

**Family composition.** We divide family compositions into three categories: single, pair or couple and family with dependants. We categorize families based on the number of adults, children and other adults staying in the household.

**Table:** 

single	couple/pair	family with dependants
57	81	687

**Type of housing.** We categorize the primary dwelling of the family as ger or bashin. While this information was readily available from the survey, decisions had to be made about singular households living on a plot with multiple dwellings. In this case, if a bashin was present, the primary residence was listed as a bashin.

**Table:** 

ger	bashin
375	450

**Type of dwelling transition.** We categorize households based on what sort of dwelling they started in Ulaanbaatar with (ger or bashin) and what sort they are currently living in, yielding four transition categories: ger to ger, ger to bashin, bashin to ger and bashin to bashin. The first dwelling is taken from the residential history.

**Table:** 

ger–ger	ger–bashin	bashin–ger	bashin–bashin
349	264	26	186

### 5.2. Generative pipeline

In this section, we provide more information on how comparable data is generated from the SDP optimality model. As detailed in §4 and visualized in figure 3, a forward (i.e. in the direction of time) simulation is constructed in order to understand optimal strategy as manifested in the behaviour of agents. The forward simulation produces longitudinal data for each agent, logging information about the agent’s state variables as well as behaviours (build, save, move) taken in each time step t. From this longitudinal data, it is possible to extract a cross-sectional subset of agents, at various time steps, in order to have a comparable dataset to the empirical record from Ulaanbaatar.

As time in the environment is a crucial determinant of where an agent is in the state space, we use the exact time structure from the Ulaanbaatar data in our data generation process. This means we take the time each household has spent in Ulaanbaatar as the time to which to run the SDP optimality model, repeating the process for each of the 825 households we have information on. We coerce any household residence time value over 40 years to 40 years. Households that have been in the environment longer than that are considered as having run the full length of their dwelling decision-making lifetimes. We thus obtain a generated dataset with the same number of households, with equivalent residence times in the environment.

The subsequent section details how we make use of ABC to systematically compare generated and Ulaanbaatar data.

### 5.3. Approximate Bayesian computation

ABC is a likelihood-free simulation-based computational method for estimating posterior distributions of parameters. Made possible by improvements in computation capacity over the past few decades, ABC approaches rely on the comparison of reference data with data simulated from a generative model in order to identify the parameter values most consistent with the observed record. The technique is more thoroughly reviewed for evolutionary audiences in Kandler and Powell [13] and Csilléry *et al*. [70]. While relatively underexplored in human evolutionary sciences focusing on current populations, ABC has already received attention in archaeology with valuable insights [69,71–74].

We implemented the ABC algorithm using R 4.1.0 [75]. The SDP optimality model and forward simulation serve as the generative model, producing data (sim data) that can be directly compared with the empirical data collected in Ulaanbaatar (UB data). Specifically, generated data and Ulaanbaatar data are compared on several parts of the state space:

— The frequency of each of the dwelling transition categories (ger–ger, bashin–bashin, bashin–ger, ger–bashin).
— The frequency of households in each state, apart from the house state, as this is incorporated in the transition categories. Savings has two levels, tenure has two levels and family has three levels, this yields seven frequencies to compare.

The aim of the ABC analysis is to infer input parameter values that can generate model outputs that most closely resemble the empirical data from Ulaanbaatar. For this, we simplify to five parameters: p_s_save, p_l_move, p_h_build, the build condition and the final pay-off scenario. We do not include the additional loss parameters (p_s_loss, p_force_move) in the ABC analysis, as the model analysis shows they do not enact unique dynamics and rather have mirror effects to p_s_save and p_l_move, respectively (see electronic supplementary material, S2). This also computationally simplifies the ABC.

**Figure 8.**
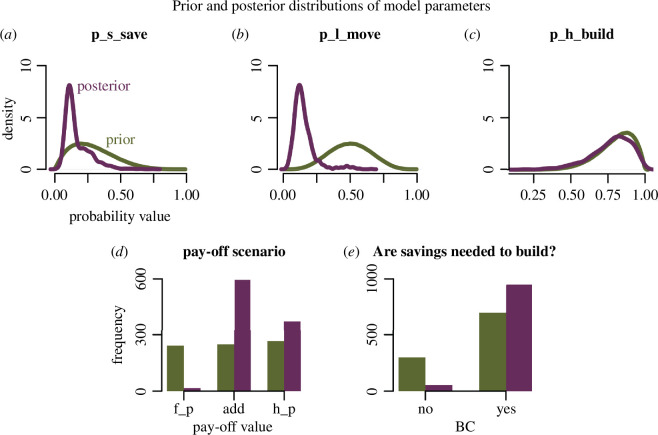
Results of ABC analysis showing parameter value representation in joint posterior: prior and posterior distributions of model parameters (panel (*a*) for parameter p_s_save, panel (*b*) for p_l_move, panel (*c*) for p_h_build, panel (*d*) for the final pay-off scenario and panel (*e*) for the build condition (BC)) obtained from 1000 samples from the joint posterior.

We first generate 1 000 000 combinations of parameter values which serve as input to the generative model. We visualize the distributions individual parameter values are drawn from (the priors) in figure 8. Given prior work in the Ger districts indicates low wealth values, our prior on p_s_save is a beta distribution peaking at 0.2, to indicate saving is unlikely. We postulated that moving results in tenure acquisition about half the time (beta prior peaks at 0.5) based on our ethnographic experience. Likewise, our ethnographic experience suggested that households building fixed houses rarely face issues, and so our beta prior for p_h_build peaks at 0.8. We included flat priors to the final pay-offs as we had no strong information to indicate which pay-off should be more likely. However, based on our ethnographic work, bashins did seem generally more expensive than gers, so our prior for build condition (BC) indicates a greater likelihood of savings being needed for building.

Subsequently, generated data (a cross-sectional dataset as described in §5.2) for each parameter combination is generated, and compared with the Ulaanbaatar data. A critical component of an ABC analysis is the sampling algorithm, which determines what level of difference between the generated data and empirical data is accepted. Commonly, rejection algorithms are used, where an optimal value is chosen, whereby any difference between the two datasets greater than that value is rejected. Given the stochasticity of our theoretical model, it is difficult to find an optimal value of difference to accept, as the strict criterion of zero difference is untenable. As such, we instead make use of importance sampling, following Fan and Sisson [76]. We save the sum difference of each of the difference frequencies detailed above. The sum of the differences is the raw output of the ABC analysis. We then construct the ABC posterior by first exponentiating the negative of the raw ABC output, to translate it to be between 0 and 1, and then normalizing it so that all of the outputs sum to 1, creating a posterior probability space. As a result, we can then sample from this posterior with a probability weighed by the ABC result. The result of the ABC analysis is thus a joint posterior, detailing values of best fit for each parameter combination.

It is important to note that an ABC analysis always generates a posterior, so the mere presence of a posterior is not diagnostic, and care must be taken in inferring what the shape of the posterior indicates. In our analysis, tight posteriors indicate more certainty on parameter values, but wide posteriors could mean both equifinality between different parameter combinations as well as a general lack of fit with the theoretical model under question. Future work and higher data resolution could unpick these problems.

**Validation.** In order to validate the ABC algorithm, the first step was to run it on synthetic data generated from the theoretical model under known parameter values, and explore how well it is able to recover the input parameter combination. The results of this validation are documented in detail in electronic supplementary material, S5. They provide a basis for trusting the technical correctness of the implementation. The validation was run on synthetic data generated for 1000 agents, for 20 parameter combinations. The ABC algorithm tested these against 100 000 parameter combinations, for 1000 agents.

The second steps in validating the ABC algorithm were posterior predictive checks. A posterior predictive check runs the whole ABC process backwards. It involves taking a sample from the ABC posterior, along with the parameter combination used to generate synthetic data that produced the ABC results, and using the parameter combination as input into the generative model. Finally, the generated data are compared with the reference data initially used in the ABC to see if the model is actually able not just to recover the parameters, but to also generate data that is comparable to the reference data. The result of the posterior predictive check for the entire validation is discussed in electronic supplementary material, S5.

A posterior prediction plot is also produced in the results to show what the ABC results imply about the dwelling transitions over time in Ulaanbaatar.

### 5.4. Results of the approximate Bayesian computation analysis

The prior and posterior distributions resulting from the ABC analysis, for each model parameter, are visualized in figure 8. The posterior distributions reflect parameter values that are most likely to produce model outcomes that are as similar as the model will allow to the reference data, the empirical record from Ulaanbaatar.

We take 1000 samples from the joint posterior and produce distributions for each of the continuous parameters (p_s_save, p_l_move, p_h_build). The density shows the frequency of values of each parameter that were present in 1000 samples of the joint posterior. For the categorical parameters, we produce bar plots which represent the frequency of each parameter value in 1000 samples from the joint posterior.

The posterior for p_s_save shows that the probability of accruing savings when saving is low, peaking at approximately 0.1 (median = 0.13, 89% highest posterior density interval (HPDI) = [0.06, 0.29]). The posterior for p_l_move shows that the probability of moving and accruing tenure is also low, likewise peaking at approximately 0.1 (median = 0.13, 89% HPDI = [0.05, 0.23]). Conversely, the posterior for p_h_build, the probability of gaining a house when building, remains at prior value, suggesting the ABC analysis was not able to recover this probability (median = 0.78, 89% HPDI = [0.60, 0.98]). The ABC validation analysis shows that the information contained in p_h_build, and to some extent p_l_move was difficult to recover (see electronic supplementary material, S6 for more detail), suggesting that the data resolution of this study was not enough to infer these parameters, a point we return to in the discussion.

The posterior values for the pay-off scenario show that the final pay-off scenario that would be most likely to produce data similar to the data collected in Ulaanbaatar is the additive scenario (59.4% of posterior samples). In this scenario, each state matters and households maximize them all. However, the house priority scenario is also identified (37.3% of posterior samples). The ABC analysis thus does not strongly distinguish between the additive and house priority scenarios. The main difference between these two final pay-off scenarios is that the house priority scenario is family state insensitive. The family priority scenario is shown to be unlikely to produce the observed data. The results suggest that each factor is important to households in Ulaanbaatar and there is uncertainty about their relative ranking.

The posterior values for the build condition suggest that savings are required for building (94.6% of posterior samples), as input parameters in the model to produce results similar to those observed in Ulaanbaatar.

While informative, looking at the marginal distributions of model parameter values is insufficient, because observable outcomes depend upon all parameters simultaneously and the parameters covary in the posterior. To explore the result on the outcome scale, we first present the inferred optimal trajectory given the ABC result.

**Figure 9.**
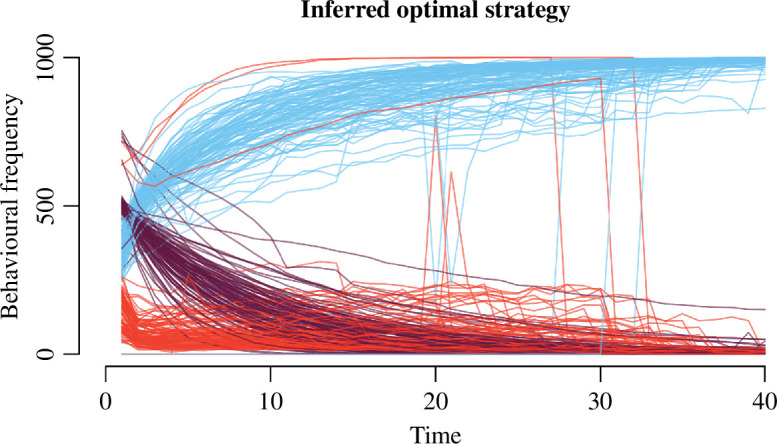
Optimal behavioural trajectory implied by ABC joint posterior: the implied optimal strategy for Ulaanbaatar given 100 samples from the ABC joint posterior. This plot echoes figure 4, showing the frequencies of building, saving and moving behaviours at each time step when 1000 agents are considered.

We take 100 samples from the ABC posterior and run these through the SDP optimality model and forward simulation for 1000 simulated households. From the outcome, we generate a plot equivalent to figure 4. The result is figure 9. The comparison between figure 4 and figure 9 allows us to see what the data suggest in terms of the optimal strategy that could produce them given the considered model.

Figure 9 matches several panels from figure 4, indicating a mix of optimal strategies represented in the posterior. Because the ABC result produces a joint posterior, draws from the posterior give us combinations in the parameter space instead of isolated values. This manifests as different optimal trajectories in figure 9. The mix of trajectories reflects the posterior distributions of individual parameters, however (as in figure 8). Figure 9 most closely resembles figure 4*f*, reflecting the additive final pay-off scenario when savings are required for building. Most households save throughout model run time, with the frequency of moving dropping after the first quarter. Building behaviour likewise peaks early on, but does not disappear completely, indicating a continued, albeit low, propensity for building well into later residence years. Some inferred optimal trajectories are better matched to figure 4*c*, however. Figure 4*c* represents an optimal trajectory of behaviour for the house priority final pay-off scenario when savings are not required. Interestingly, this indicates that when savings are not needed for building a fixed house, the more likely final pay-off scenario is house priority, which does not optimize for family state growth. In contrast, when savings are required for building a fixed house, the most likely final pay-off scenario is additive, with all states maximized.

**Figure 10.**
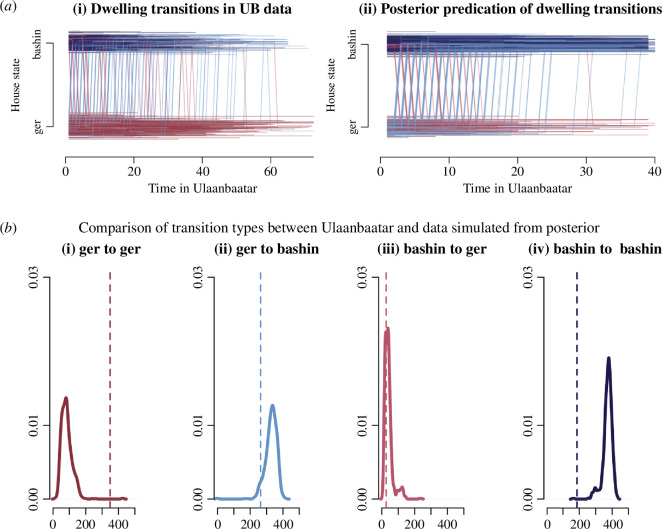
Comparison of realized and implied dwelling transition trajectories: results of ABC analysis as viewed through dwelling transitions, in comparison with empirical dwelling transitions observed in Ulaanbaatar. (*a*) Dwelling transition plots for (i) data from Ulaanbaatar, and (ii) based on a generated dataset where each observation is simulated from the generative model based on parameter values taken from the joint posterior constructed from the ABC result and run to the relevant ‘time in Ulaanbaatar’ taken from the reference data. Households (lines) are categorized based on the type of dwelling transition: ger–ger (dark red), ger–bashin (light blue), bashin–ger (light red), bashin–bashin (dark blue). The x-axis represents time in environment, while the y-axis represents the type of dwelling a household is in, ger or bashin. (*b*) Frequencies of each dwelling transitions type. Distributions indicate values from 1000 datasets generated from 1000 draws from the joint posterior. Dashed line indicates frequency from Ulaanbaatar data.

We also explore the implications of the joint posterior for dwelling transitions, as represented by changes in the house state. We generate a dataset comparable to the data collected in Ulaanbaatar (825 observations, and with the same population structure for time in Ulaanbaatar). For each observation, we take a sample from the joint posterior and use this as input for the generative model. The model is then run to the relevant time in Ulaanbaatar to generate state values. We then use this generated dataset, which encapsulates variation in the posterior, for comparison to data on dwelling transitions and their timing in the Ulaanbaatar data, yielding figure 10*a*. Figure 10*a* can be directly compared with figure 5.

Comparing actual dwelling transition trajectories from the Ger districts (panel (i)) with those of the posterior prediction (panel (ii)) in figure 10*a*, shows that the posterior prediction captures the high density of dwelling transitions in early residence years and also captures that both types of transitions, ger–bashin and bashin–ger are occurring, even in later years. However, there are discrepancies in the types of dwelling transitions that the model captures. We produce figure 10*b* which compares the frequencies of dwelling transitions categories (ger–ger (dark red), ger–bashin (light blue), bashin–ger (light red), bashin–bashin (dark blue)) between the empirical data and posterior prediction. The model captures bashin to ger transitions and ger to bashin transitions, but fails to account for stability, missing the frequencies of ger to ger and bashin to bashin transitions. The posterior prediction over-represents households in bashins and under-represents households in gers, suggesting a mechanism for households staying in gers is missing from the model.

### 5.5. Discussion of empirical case study

We have followed a generative inference approach to explore housing decisions in the Ger districts of Ulaanbaatar. Using the theoretical model developed in §4, we have analysed transitions between different dwelling types in relation to economic and demographic factors of households. Based on how well our theoretical model is able to predict the empirical data observed in Ulaanbaatar, we can conclude that the situation in Ulaanbaatar’s Ger districts is consistent with a optimality scenario in which households optimize for housing, tenure, as well as household capital, and family advances to a lesser degree. Likewise, our analysis suggests housing costs are high in the Ger districts, moving rarely results in obtaining tenure, but once households build they do transition from a ger to a house.

In this discussion, we focus on two points: firstly, the deviation from optimal as presented by the persistence and number of households in gers; secondly, in terms of thinking through how we would address a mix of strategies in a population.

The length of time and number of households that remain in gers and bashins is underestimated by the model, in relation to the empirical data. This suggests we are missing a mechanism that would allow households to remain in the dwellings they start with, especially gers. Firstly, it is possible that households remain so not by choice; they are not able to pursue optimal trajectories. From our interviews, it is clear that bashins are seen as ‘the thing done’ in the Ger districts, and so when households do not build bashins, it may reflect a real inability to obtain enough resources or support to build. This is also reflected in the ABC posterior, which suggests savings are difficult to accrue in this environment. However, as the posterior predictions suggest, these probabilities are not enough to account for the number of households in gers. That being said, the work presented here cannot evaluate the potential role for poverty traps or other wealth-material feedbacks that may in fact make it much more difficult for households to change than postulated by the model. Additionally, in the current version, agents are initialized randomly into the state space, but most households coming into the ger districts start with a ger—this discrepancy warrants further research.

Alternatively, staying in a ger may reflect an unconsidered optimal behavioural strategy. In addition to gers and bashins, households can also choose to move to a central city apartment, leaving the ‘environment’ altogether. Our model does not capture a situation like this, whereby households may choose to save up and stay in low-cost housing in order to wait for an opportunity to leave the Ger districts, thus extending their ger time. Modelling such behaviour would also require a more complex treatment of mobility, for purposes other than gaining tenure. Given also the literature on use time and intended use time in both the TIM and landscape investment research [26,30,31], our approach would benefit from an explicit inclusion of intended stay. Unfortunately, assessing intended stay against realized behaviour would require longitudinal data, increasing the practical burden of this work.

Finally, we return to the optimality premise in the conclusion of this manuscript. Optimality is a property of *model trajectories*, but understanding if behaviour is optimal in the real world is a separate concern.

Figure 9 highlights how the parameters of the model do not work in isolation. Viewed on the outcome scale of the implied frequency of behaviours over time, the most represented strategy is in line with the additive pay-off scenario, with savings required for building. A smaller portion of the outcome also more closely resembles the house priority scenario with savings not required for building. This mix of strategies, so to speak, may suggest differences in the population in terms of what is being optimized, and under what conditions.

Covariances in the parameter space urge us to think more explicitly about differences in the population in relation to optimal strategies and end goals in particular. Households that have no intention of staying in Ulaanbaatar are certainly going to have different strategies to households that aim to make it their permanent home. Likewise, the Ger districts have a rich age mix [65]. Many retired parents choose to follow their young adult children to the city, often with explicit aims to help with future childcare needs. Their housing strategy is likely to be different from young families. Indeed, there may also be differences between households in terms of experience with urban environments, whether encoded in migration histories or birthplaces. Future work could thus focus on a more relational approach, connecting state space variables in more nuanced ways. Of course, the trade-off between model simplicity and validity will always be a key concern.

Finally, a few methodological points to consider. The SDP model was built based on theoretical considerations. Simulating optimal trajectories from the SDP model shows that some scenarios have little difference between them—the simulation variance within a parameter combination is not appreciably smaller than the simulation variance between parameter combinations for certain areas of the parameter space. While this is a result in itself, indicating that theoretically justified differences do not result in differences in optimal trajectories, particularly in dwelling transitions, it is important to keep in mind when considering the ABC results. ABC analyses are valuable in that they explicitly uncover situations of equifinality [13]. As in this study, the result for which pay-off scenario, additive or house priority, is being optimized, is not clear cut. However, the joint posterior reveals patterns of association that can help formulate future research. In this case, the result that a house priority pay-off scenario is likely to be associated with the lack of a need of savings for building, comes directly from the posterior prediction. An important advantage of a generative inference approach is that deviations from model predictions are meaningful and can help structure future research. Combined with transparent analysis and available data, we argue this approach scaffolds future work.

In order to conduct the ABC analysis, it is necessary to simulate data from the SDP model that matches the empirical record. In these situations, it is possible that the resolution of our data is not high enough to infer all the parameters through the ABC analysis. The ABC validation shows that specifically the p_h_build and to some extent also p_l_move parameters were difficult to recover in our set-up. Issues of resolution, while sometimes insurmountable, can motivate use of generative inference tools in data collection planning and validation. In this way, it is possible to assess exactly what sort of data is needed to infer parameters of interest before data are collected.

Our research echoes past work on housing investments, putting the costs of dwellings (here in terms of savings, not time [31]) centre stage. In the model we present, a key difference in the optimal trajectory is brought on by differences in whether savings are required to build or not. As such, understanding the material investments required in dwellings is paramount to understanding how, and why, households change dwellings or not. We do not model maintenance costs that are likely to differ systematically between the two kinds of dwellings. Given the importance of the investment trajectory, not just decisions at a single point in time, future work needs to include running costs.

Likewise, more ethnographic and descriptive work is required to understand how exactly initial and maintenance costs relate in the dwelling context. For example, in the Ger districts and in other urban environments across Mongolia, an urban ger is not the same as a steppe ger: steps are taken to make its construction suitable for sedentary urban life. A concrete platform is often constructed to provide better insulation and a stable floor, while an entrance room is constructed for storage and insulation [60]. Such investments may be cheap in absolute terms, but can still influence decision-making about mobility and future investment choices, particularly in resource scarce situations. While a major difference between gers and bashins is that bashins have higher front-loaded investment, requiring upfront investment for construction, maintenance and long-term improvements to either bashin or ger may also create ‘high investment’ dwellings, but the investment occurs over a longer period of time.

There is a difference between a high-investment dwelling with lots of front-loaded investment and a high-investment dwelling that has been made so through years of adjustment and improvement.

By emphasizing the importance of a detailed understanding of costs, our research motivates ways to connect settlement strategy again with ecology. The materials populations need for appropriate construction can come under the lens of evolutionary modelling, contributing to a comparative and time-sensitive understanding of human–environment relationships.

## 6. Conclusion

In this study, we have followed a generative inference approach to understand household settlement strategy; the decisions households make about where to live, for how long and what to build there.

The generative inference approach requires greater engagement with the mechanics of a system, relying to a much greater extent than standard data analysis on a well-specified generative model that makes explicit assumptions about the causal processes at play.

To this end, we have developed a theoretical model of housing decisions, building on the long history of human behavioural ecology work on human–environment interactions that makes use of optimality models to generate directional predictions and test these with empirical data from contemporary populations to unpack deviations from model scenarios.

Our theoretical model has sought to explore the trade-offs between moving, building and saving behaviour over a lifetime horizon, to understand how these behaviours could be structured over time to achieve different optimization goals. We have also focused on the costs of different dwelling types and their interaction with optimization goals and behavioural trajectories. By explicitly modelling housing as a state variable, we implicitly include behavioural feedbacks from the built landscape.

We find that in circumstances where housing changes are costly, saving dominates early on to allow for future building behaviour. Mobility functions to obtain tenure in our model and thus, depending on the value of tenure in the final pay-offs, generally takes place early on. We also find scenarios where stochasticity plays an important role in optimal behavioural trajectories, causing bifurcations in optimal trajectories.

Additionally, viewing optimal strategies through the lens of dwelling transitions, or dynamics in the house state, shows that transitions are bi-directional. Thus, it can be optimal to ‘lose’ house state, with important implications for understanding long-term changes in land use.

It is tempting to see these conclusions as obvious implications of model assumptions. However, while they are indeed logical implications of the model assumptions, they are not necessarily obvious. Many logical results appear obvious post hoc, but are opaque prior to computation. But even if they were obvious, they must still be demonstrated logically, as we have done.

We have subsequently used our model as an analysis tool, comparing synthetic data simulated from the model with empirical data collected in the Ger districts of Ulaanbaatar to understand household dwelling transitions in relation to settlement strategy. The aim of this analysis was to derive the closest match: given the model, what parameter combinations are most likely to produce data such as that observed in the empirical case.

Our results suggest the model does well in accounting for the spread of dwelling transitions over time, and the bi-directional nature of transitions. The main deviation from model predictions comes from the number of households not changing dwellings, specifically the number of households remaining in gers. This deviation suggests our model is missing a mechanism that would allow households to stay in mobile dwellings. We have discussed possible avenues of extension in the discussion section of our empirical case and would like to stress here that it is only because of directional predictions from our model that we are able to productively address how to move forward. In the absence of explicit generative models, null results hold little value for scaffolding future research.

Our theoretical model is by definition an optimality model, as it derives optimal trajectories of behaviour to achieve pre-specified final pay-offs. However, it is important to state that the assumption of optimality remains in the model world; it makes no claims of, and does not test, optimality in the real world. The optimality assumption is pragmatic; it aids our theoretical understanding and provides a clear set of predictions against which to assay real-world behaviour. Crucially, it provides a framework for interpreting deviations from model predictions, which can subsequently be used to improve the model and thus our understanding.

To conclude, we contribute to an evolutionary understanding of constructed landscapes by calculating optimal trajectories of behaviour over time, looking at trade-offs between moving, building and saving decisions. By explicitly modelling dwellings in the state space, we were implicitly able to integrate feedbacks from the built environment in optimal trajectories.

Understanding how decision-making at the household level scales up to settlement dynamics needs to consider the construction of the landscape as an active component. We have begun by exploring this dynamic through the lens of residential dwelling transitions. This view is necessarily a simplification, and we hope future research considers other aspects of the settlement landscape, particularly feedbacks from other households, to expand our understanding. But even more simply, our work suggests we need to provide descriptive accounts of the longevity and costs of certain forms of housing, and the impact they have on the landscape and human livelihoods.

Regardless of the specific contributions of this paper, future progress in understanding the evolution of the built environment depends upon the integration of explicit coevolutionary models and statistical techniques that implement those theories directly to produce estimates. In this light, an additional contribution is a fully documented example of a framework for implementing, validating and fitting dynamic and strategic models of human–environment coevolution. This kind of framework is useful far beyond the specific topics of this paper, and we hope the materials provide a foundation for others to both extend our work and to take it in entirely new directions.

## Data Availability

All code and materials required to run the optimality model and its analysis are available here: [77]. The data required for the empirical analysis are available online [78]. Supplementary material is available online [79].
